# Intermittent hypoxic pretreatment exacerbates house dust mite‐induced asthma airway inflammation

**DOI:** 10.1002/iid3.1253

**Published:** 2024-04-17

**Authors:** Hao Meng, Dongxue Zhang, Yifan Que, Peng Hu, Runsheng Wang, Yunfei Liao, Guogang Xu

**Affiliations:** ^1^ Health Management Institute, The Second Medical Center & National Clinical Research Center for Geriatric Diseases, Chinese PLA General Hospital Medical School of Chinese PLA Beijing China; ^2^ Department of Endocrinology, Beijing Shijitan Hospital Capital Medical University Beijing China; ^3^ Department of Respiratory and Critical Care Medicine, The Second Medical Center & National Clinical Research Center for Geriatric Diseases, Chinese PLA General Hospital Medical School of Chinese PLA Beijing China; ^4^ Department of Endocrinology, Union Hospital, Tongji Medical College Huazhong University of Science and Technology Wuhan China; ^5^ Hubei Provincial Clinical Research Center for Diabetes and Metabolic Disorders Wuhan China

**Keywords:** asthma, intermittent hypoxic preconditioning, NF‐κBTh2 inflammatory response

## Abstract

**Background:**

Asthma is widely recognized as an inflammatory disorder. In the context of this inflammatory microenvironment, the involvement of hypoxia and its impact on related pathways have drawn considerable attention. However, the exact role of hypoxia, a prevalent environmental factor, in the development and progression of asthma remains poorly understood.

**Methods:**

Mice were treated with house dust mite (HDM) extracts for 23 days to induce asthma. Mice were divided into room air (RA) group and intermittent hypoxic (IH) group by exposing to different conditions and IH preconditioning (IHP) were underwent to the above groups before the hypoxic regimen. Airway inflammation in mice was evaluated by airway hyperresponsiveness, excessive mucus secretion, and recruitment of inflammatory cells. Immunohistochemistry was employed to quantify the expression levels of NF‐κB. Subsequently, the dose of allergen was modified to investigate whether the impact of hypoxia on asthma is affected by different doses of allergens.

**Result:**

Compared to the RA and IH groups, HDM‐treated mice in the IHP group exhibited aggravated inflammatory cell infiltration and airway hyperresponsiveness (*p*＜.05). Moreover, there was an increased release of inflammatory mediators and higher expression levels of NF‐κB (*p*＜.05). Importantly, the impact ia on asthma was found to be influenced by high dose of allergen (*p*＜.05).

**Conclusion:**

IHP treatment potentially exacerbates HDM‐induced airway inflammation in asthma, with the involvement of NF‐κB, particularly under high‐dose allergen stimulation.

## 1 INTRODUCTION

Asthma, a complicated and heterogeneous disease,^1^ represents a chronic respiratory disorder influenced by multiple environmental factors.^2^ It is usually characterized by predominant Th2‐type inflammation, leading to airway remodeling and increased eosinophilic infiltration.^3^, ^4^ A recent research has indicated that the prevalence of asthma among Chinese adults is 4.2%,^5^ significantly impairing the health of tens of millions individuals. Asthma has emerged as a substantial burden on the economic and social development in China.

In pulmonary diseases, compromised ventilation dynamics, airway obstruction, alveolar exudates, interstitial thickening due to edema, inflammation, fibrosis, or damage to pulmonary capillaries in the alveoli can result in significant and potentially life‐threatening impediments to normal oxygenation.^6^ Simultaneously, pulmonary diseases are also influenced by hypoxia. Oxygen deficiency induces endothelial damage and functional impairment, leading to increased permeability, heightened vascular smooth muscle tension, and cellular proliferation—all of which play roles in tissue remodeling processes and inflammatory responses.^7^

In the realm of pulmonary diseases, hypoxia exerts a multifaceted impact. In acute lung injury (ALI), hypoxia exacerbates inflammation through the Toll‐Like Receptor 4 Signaling Pathway.^8^

Upregulation of mitochondrial autophagy mediated by FUNDC1 activates the reactive oxygen species (ROS)‐HIF1α pathway, promoting pulmonary artery smooth muscle cell proliferation and consequently leading to pulmonary arterial hypertension.^9^ Chronic obstructive pulmonary disease (COPD) patients at high altitudes, experiencing chronic oxygen deprivation, exhibit markedly restricted exercise endurance, intensified breathlessness, and hypoxemia.^10^ The expression of hypoxia‐inducible factor (HIF‐1), induced by hypoxia, upregulates the expression of inflammatory factors by activating the EGFR/PI3K/AKT pathway, thereby worsening COPD.^11^ Similarly, in COPD, ROS generated due to hypoxia, as an intrinsic stressor, induce myoblast pyroptosis through the NF‐KB/HIF‐1α pathway.^12^

Certain diseases characterized by hypoxia symptoms may exacerbate inflammatory manifestations in asthma. Of particular note, patients with both COPD and asthma exhibit higher rates of exacerbation, hospitalization, incidence, and mortality compared to individuals with either asthma or COPD alone.^13^ The coexistence of obstructive sleep apnea syndrome not only serves as an independent risk factor for asthma worsening but also intensifies asthma symptoms.^14^ The presence of chronic sinusitis in asthma patients increases the frequency of asthma attacks while simultaneously impairing symptom control and life quality.^15^ Based on the evidence above, we proposed a hypothesis that hypoxia associated with these comorbidities maybe contribute to poor asthma control. Hypoxia may lead to more severe airway hyperresponsiveness and inflammatory responses in asthma.

The interplay between hypoxia and inflammation is of paramount significance, with numerous reports indicating that hypoxia exacerbates existing inflammation. However, the impact of low oxygen levels on diseases and inflammation is not a simple intensifying effect; rather, it involves a complex regulatory role. Hypoxia signaling can exhibit both anti‐inflammatory and proinflammatory effects, thereby modulating the activity of immune system cells.^16^ While hypoxia may promote inflammation and lead to the disruption of mucosal and vascular barriers, adaptive responses to hypoxia, particularly the stabilization of HIFs, confer anti‐inflammatory and tissue‐protective effects.^17^

The impact of hypoxia on asthma remains a subject of divergent perspectives. Some studies propose that low oxygen levels exert adverse effects on asthma, intensifying airway inflammatory response.^18^, ^19^, ^20^, ^21^ Chronic intermittent hypoxia enhances the activation of the p38 MAPK pathway, oxidative stress damage, and NF‐κB expression in lung tissues, thereby exacerbating pulmonary inflammation.^18^ Hypoxia can influence CD8+ type 2 cytotoxic T cells via HIF‐α, leading to increased IL‐13 secretion and worsening asthma.^19^ In mouse models, hypoxia intensifies allergen‐induced HIF‐1α, chemokines, airway inflammation, TGF‐β1, and airway remodeling.^20^ Additionally, hypoxia exacerbates asthma through involvement in the renin‐angiotensin system, contributing to epithelial‐to‐mesenchymal transition and airway remodeling.^21^ However, contrasting findings suggest that hypoxia can mitigate allergic airway inflammation and improve airway remodeling, yielding beneficial effects on asthma.^22^, ^23^, ^24^ Low oxygen levels induced human umbilical extracellular vesicles from cord mesenchymal stem cells, demonstrating the potential to alleviate allergic airway inflammation and airway remodeling in a chronic asthma mouse model.^22^ Hypoxia further alleviates allergic asthma by downregulating the MAPK signaling pathway.^23^ Hence, the impact of hypoxia on allergic asthma inflammation and airway reactivity is a topic marked by divergent viewpoints, necessitating further data and pathway research.

HIF‐1α, a pivotal dimeric protein complex, plays an indispensable role in the human response to low oxygen concentrations, serving as a primary regulatory factor in hypoxic reactions.^25^ NF‐κB, a crucial transcription factor required for the expression of various proinflammatory molecules, holds a vital role in lung inflammation in conditions such as asthma.^26^, ^27^ Functioning as a direct regulator of HIF‐1α expression, NF‐κB modulates the levels of HIF‐1α.^28^, ^29^ Thus, NF‐κB, being a shared target in the response to low oxygen and inflammatory reactions, is likely involved in the exacerbation of pulmonary inflammation in asthma under hypoxic conditions, although conclusive evidence is currently lacking.

This study aims to investigate the impact of hypoxia on a mouse model of asthma, delving into aspects such as symptom control, TH2‐type immune responses, and the types of inflammatory cells. Furthermore, the study seeks to explore the potential involvement of NF‐κB in mediating these effects.

## 2 MATERIALS AND METHODS

### 2.1 Animal and allergen

**Figure 1:**
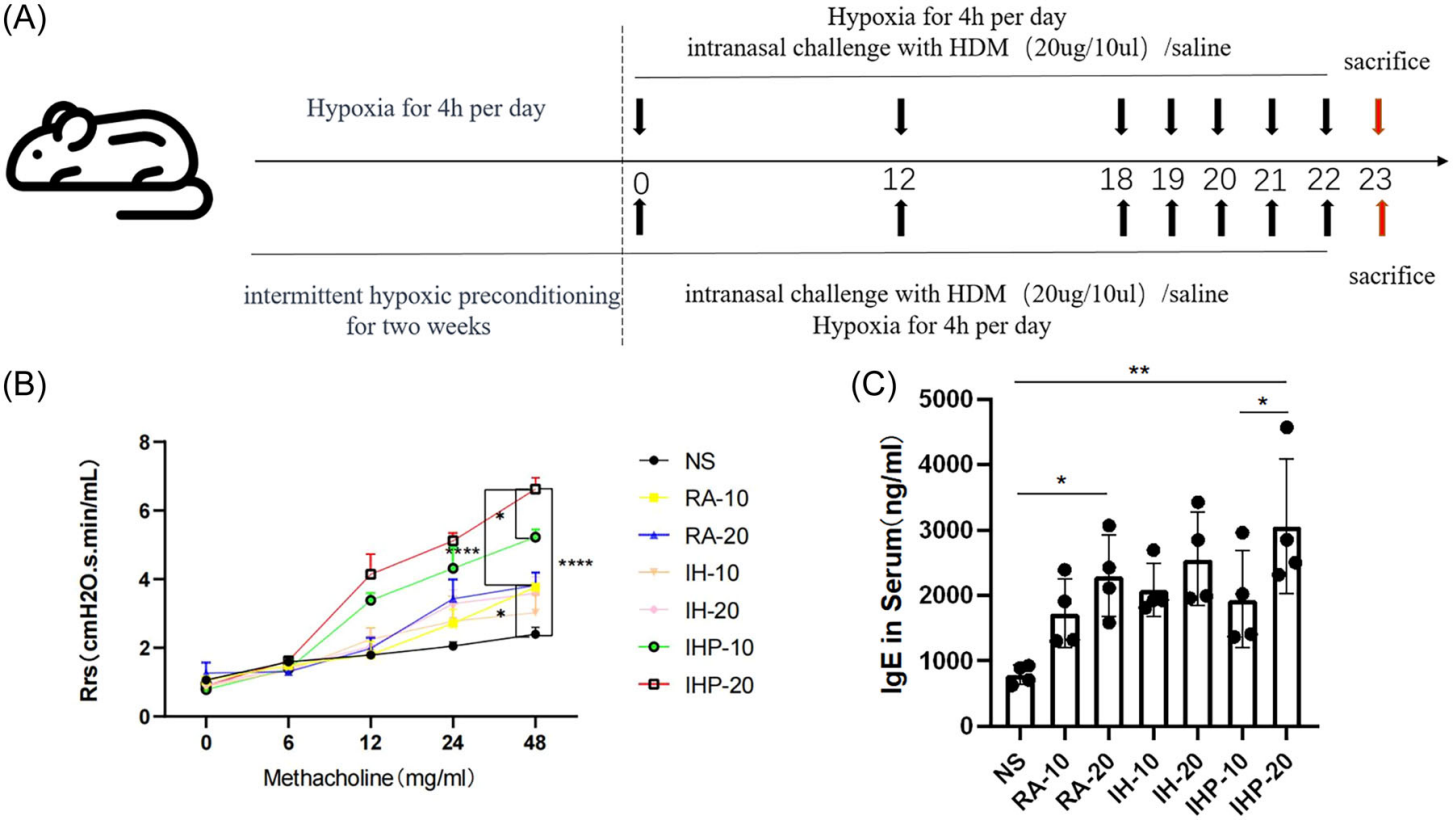
Resistance of the respiratory system and serum IgE levels in asthma. (A) Protocol of animal experiments. NS: normal saline in room air; RA‐10: HDM (10 μg/50 μL) treatment in room air; RA‐20: HDM (20 μg/50 μL) treatment in room air; IH‐10: HDM (10 μg/50 μL) treatment in intermittent hypoxia exposure; IH‐20: HDM (20 μg/50 μL) treatment in intermittent hypoxia exposure; IHP‐10: HDM (10 μg/50 μL) treatment in intermittent hypoxia with a 2‐week Intermittent hypoxic pretreatment. IHP‐20: HDM (20 μg/50 μL) treatment in intermittent hypoxia with a 2‐week Intermittent hypoxic pretreatment. (B) Measurement of airway hyperresponsiveness. (C) Total IgE in serum. (*n* = 4 in each group) *p < .05, **p < .01, ***p < .001, ****p < .0001.

Female BALB/c mice aged 6−8 weeks, weighing 20−22 g, were obtained from Beijing Vital River Laboratory Animal Technology Co., Ltd. They were randomly divided into experimental and control groups and housed in the SPF‐grade barrier animal facility at the Animal Experimental Center of Capital Medical University, Beijing, China. The mice were maintained under a 12‐h light‐dark cycle at a temperature of 25°C and were housed in a specific pathogen‐free environment. The detailed method of model construction was described as Figure 1A. House dust mite (HDM) extract, purchased from Cosmo Bio Co., Ltd., with product code LSL‐LG‐5339 was used as allergens in this research.

### 2.2 Animal model construction

BALB/c mice were intranasally sensitized with HDM (20 μg in 50 μL PBS) on Day 0 and 12, followed by intranasal stimulation with the same allergen from Day 18 to 23 to establish the asthma model. The room air (RA) group was exposed to RA for modeling, the IH group was modeled under intermittent hypoxia conditions (4 h/day), and the IH preconditioning (IHP) group underwent intermittent hypoxia preconditioning for 2 weeks before modeling under IH conditions (4 h/day). The low‐dose group received a reduced HDM dose of 10 μg in 50 μL PBS, while the remaining procedures were the same. The hypoxia induction protocol involved placing mice in a low‐oxygen chamber for 4 h daily, with an oxygen concentration of 10%. Subsequently, the mice were returned to RA with an oxygen concentration of 21%. The mice models are divided into the following seven groups: the NS group received intranasal normal saline treatment in RA; the RA‐10 group received intranasal HDM (10 μg/50 μL) treatment in RA; the RA‐20 group received intranasal HDM (20 μg/50 μL) treatment in RA; the IH‐10 group received intranasal HDM (10 μg/50 μL) treatment in intermittent hypoxia exposure; the IH‐20 group received intranasal HDM (20 μg/50 μL) treatment in intermittent hypoxia exposure; the IHP‐10 group received intranasal HDM (10 μg/50 μL) treatment in intermittent hypoxia exposure with a 2‐week Intermittent hypoxic (IH) pretreatment; and the IHP‐20 group received intranasal HDM (20 μg/50 μL) treatment in intermittent hypoxia exposure with a 2‐week IH pretreatment (Figure 1A and Table 1).

**Table 1:** Animal model grouping.

Group	Number of mice	gender	Modeling environment	Nasal irritants	intermittent hypoxic preconditioning	Number of nasal drops	Modeling days
NS	4	Female	Room air	Saline	No	7	23
RA‐10	4	Female	Room air	HDM	No	7	23
RA‐20	4	Female	Room air	HDM	No	7	23
IH‐10	4	Female	Intermittent hypoxic	HDM	No	7	23
IH‐20	4	Female	Intermittent hypoxic	HDM	No	7	23
IHP‐10	4	Female	Intermittent hypoxic preconditioning	HDM	No	7	37
IHP‐20	4	Female	Intermittent hypoxic preconditioning	HDM	No	7	37

### 2.3 Measurement of airway hyperresponsiveness

Mice were anesthetized with a 2% sodium pentobarbital solution at a dose of 80 mg/kg body weight administered via intraperitoneal injection. Following anesthesia, mice were positioned in a supine position on a dissecting board, and their limbs and mouths were secured. After disinfecting the neck, chest, and abdomen with 75% alcohol, a tracheostomy was performed, and a small animal tracheal tube was inserted. Tweezers were used to lift the mouse fur, and scissors were used to incise the neck skin. Blunt dissection of muscles and connective tissues exposed the trachea. A T‐shaped incision was made with ophthalmic scissors, and a tracheal tube needle was inserted. The needle was secured with sutures, and the tracheal tube was connected to a animal pulmonary function measurement system (FlexiVent). and their respiratory resistance was measured under increasing concentrations of acetylcholine (0, 6, 12, 24, and 48 mg/mL), with the results recorded and expressed as resistance of the respiratory system (Rrs).^30^ Throughout the Rrs measurement process, the respiratory condition of mice was continuously monitored, and no signs of respiratory distress were observed. Statistical analysis of the results was conducted using GraphPad Prism 8.0 software, employing the Two‐Way Analysis of Variance (ANOVA) statistical method.

### 2.4 Total cell count in the bronchoalveolar lavage fluid (BALF)

The method involved connecting a 1 mL syringe needle to the tracheal tube needle for endotracheal intubation. Subsequently, 0.8 mL of PBS was aspirated using a 1 mL syringe for intratracheal injection, followed by three repeated washes, and the collected fluid was transferred into a centrifuge tube. This process was repeated twice for each mouse, resulting in a total collection of 1.6 mL of BALF. Subsequently, the BALF was centrifuged at 1500 rpm for 10 min. After removing the supernatant, the BALF cells were treated with red blood cell lysis buffer for 4 min. The cells were then centrifuged again at 1500 rpm for 10 min, and after discarding the supernatant, they were resuspended in 500 μL of PBS. A volume of 10 μL of the resuspended cells was taken and counted using a TC20 automated cell counter (Bio‐Rad Laboratories, Inc., Hercules, CA) to determine the total cell count.^31^

### 2.5 Lung histology

After euthanizing the anesthetized animals, the left lung was excised and fixed in 10% formalin for 24 h. Subsequently, the tissue was dehydrated, embedded in paraffin, and sectioned (4 mm thick).^32^ Inflammatory changes were evaluated by performing hematoxylin and eosin (H&E) staining. The blinded scoring system described in previous literature was employed to assess peribronchial inflammation and cellular infiltration. Briefly, the scoring system ranged from 0 to 5, where 0 indicated no cells, 1 represented a few cells, 2 indicated a single layer of cells around the bronchi, 3 denoted a single layer of cells extending 2−4 cells deep, 4 indicated a single layer of cells extending 4−6 cells deep, and 5 represented a single layer of cells extending more than 6 cells deep.^33^

### 2.6 PAS staining of lung tissue

The paraffin‐embedded lung tissue was sectioned (4 mm thick) and subsequently subjected to dehydration in alcohol. Periodic acid‐Schiff (PAS) staining was performed following the manufacturer's instructions (purchased from Solarbio, catalog number G1281). The assessment of mucus secretion in lung tissue was conducted based on the scoring system described in previously reported literature.^32^ The PAS staining scoring criteria were as follows: Each airway was scored based on the percentage of surrounding goblet cells. A score of 0 represented ≤5% goblet cells, a score of 1 indicated 5‐25% goblet cells, a score of 2 denoted 25‐50% goblet cells, a score of 3 indicated 50‐75% goblet cells, and a score of 4 represented ≥75% goblet cells.^32^

### 2.7 Congo red staining of lung tissue

The previously sectioned lung tissue (4 mm thickness) was subjected to Congo Red staining using a commercially available kit (purchased from Solarbio). The staining procedure was performed according to the manufacturer's instructions. Quantification of Congo Red staining was conducted by counting the number of eosinophilic cells in 10 peribronchial units per lung lobe of each mouse and calculating the average, which represents the Congo Red count for that particular mouse.^32^

### 2.8 Measure of total serum IgE

Serum samples were collected from experimental animals and processed according to the procedure described previously.^34^ The total IgE concentration in the serum was measured using a commercial ELISA kit (Invitrogen, Vienna, Austria) following the manufacturer's instructions.

### 2.9 Measurement of cytokines in lung tissue

The lung tissues from mice were prepared as tissue homogenates using a method previously reported.^31^ Briefly, the right lung tissue was placed in a PBS solution containing 1% Triton X‐100 and protease inhibitors. After centrifugation at 10,000*g* for 10 min, the supernatant was collected. The levels of cytokines (interleukin‐4 [IL‐4], interleukin‐6 [IL‐6], tumor necrosis factor‐alpha [TNF‐α], interferon‐gamma [IFN‐γ], interleukin‐5 [IL‐5], interleukin‐13 [IL‐13], and interleukin‐17A [IL‐17A]) in the lung tissue homogenates were measured using commercial ELISA kits (Invitrogen, Vienna, Austria) according to the manufacturer's instructions.

### 2.10 Lung immunohistochemistry

Immunohistochemical staining was conducted to assess the immunoreactivity of NF‐κB by employing recombinant Anti‐NF‐κB P65 antibody (Abcam, Hong Kong, China) and secondary antibody, goat anti‐mouse IgG. Semi‐quantitative analysis was performed according to established protocols described in previous literature.^35^ Before incubating the primary antibody, block with goat serum for 30 min at room temperature to reduce nonspecific staining. Subsequent counterstaining was performed using hematoxylin, where circular cell nuclei appeared blue, enabling nuclear localization.

### 2.11 Statistical analysis

Data were presented as mean ± standard deviation. Statistical analyses were performed using GraphPad Prism 8.0 software (GraphPad). The distribution of data in each group follows a Gaussian distribution, indicating the suitability of parametric tests. One‐Way Analysis of Variance (ANOVA) was used to examine the variability among two or more groups of data originating from the same variable. On the other hand, for analyses involving two different categorical independent variables, Two‐Way ANOVA was employed. For all experiments, a *p* value less than .05 was considered statistically significant. The Tukey's post hoc test was subsequently employed for further examination of the results to determine which groups exhibited significant differences. The data from Measurement of airway hyperresponsiveness tests were subjected to Tukey's post hoc test. The results revealed that the most pronounced differences among groups were observed at the concentration of 48 mg/mL of acetylcholine, with statistical significance. The *p* values obtained from this study indicate differences in airway resistance under acetylcholine stimulation at the concentration of 48 mg/mL.

## 3 RESULT

### 3.1 IHP increases resistance of the Rrs and serum IgE levels in asthma

Mice exposed to HDM in RA exhibited an increased resistance of the Rrs relative to the control group (RA‐20 vs. NS, *p* < .05) (Figure 1B) and a rise in serum IgE levels (RA‐20 vs. NS, *p* < .05) (Figure 1C). Furthermore, the increase in resistance of the Rrs was more pronounced in the group that underwent intermittent hypoxia preconditioning (IHP‐20 vs. NS, *p* < .0001) (Figure 1B), as was the increase in serum IgE levels (IHP‐20 vs. NS, *p* < .01) (Figure 1C). Compared to the RA‐20 group, the IHP‐20 group showed a significant increase in Rrs (IHP‐20 vs. RA‐20, *p* < .0001) (Figure 1B), while the serum IgE levels showed a slight increase but without statistical significance (Figure 1C).

### 3.2 IHP exacerbates eosinophilic cell‐predominant lung inflammation in asthma

**Figure 2:**
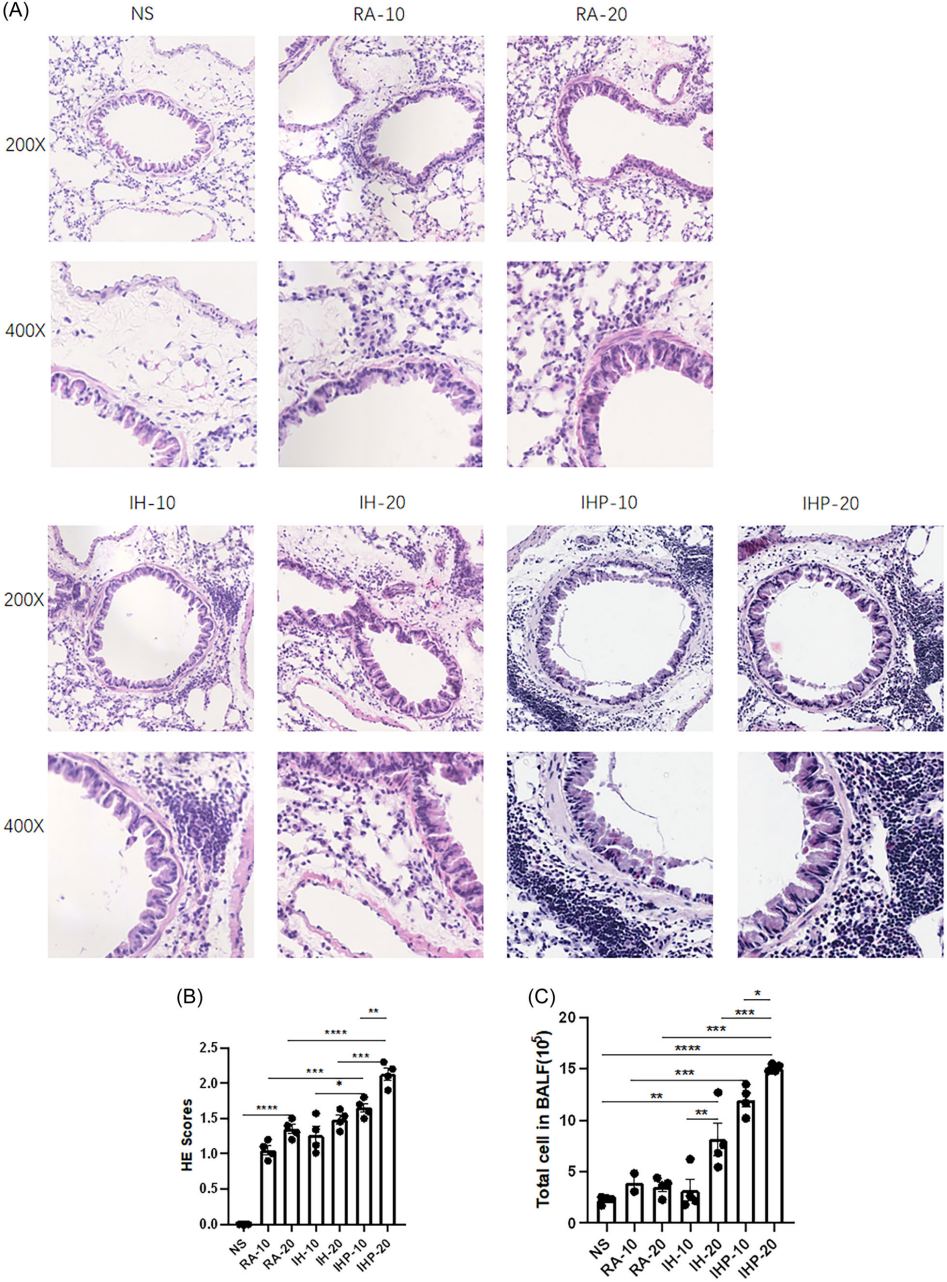
Assessment of airway inflammation. (A) Representative photomicrographs of H&E stained sections of lung tissues (original magnifications ×200 and ×400). (B) Scores of the severity of inflammatory cellular infiltration into the airways. (C) Cell counts per mL of the bronchoalveolar lavage fluid (BALF) of the groups of mice. (*n* = 4 in each group) *p < .05, **p < .01, ***p < .001, ****p < .0001.

HE staining revealed that mice sensitized to HDM in RA displayed an increased infiltration of inflammatory cells around bronchioles and blood vessels compared to the control group (RA‐20 vs. NS, *p* < .0001) (Figure 2A,B). No significant difference in scoring of HE staining was observed between the IH‐20 and the RA‐20. However, the intermittent hypoxia preconditioning group exhibited a more pronounced aggregation of inflammatory cells compared to the RA group (IHP‐20 vs. RA‐20, *p* < .0001; IHP‐10 vs. RA‐10, *p* < .05), and the aggregation of inflammatory cells was even more evident in the hypoxia preconditioning group than in the intermittent hypoxia group (IHP‐20 vs. IH‐20, *p* < .001; IHP‐10 vs. IH‐10, *p* < .05) (Figure 2A,B). Similar observations were made in the BALF, with a significantly higher total number of cells in the hypoxia preconditioning group (IHP‐20 vs. RA‐20, *p* < .001; IHP‐20 vs. IH‐20, *p* < .001; IHP‐10 vs. RA‐10, *p* < .001; IHP‐10 vs. IH‐10, *p* < .001) (Figure 2C).

**Figure 3:**
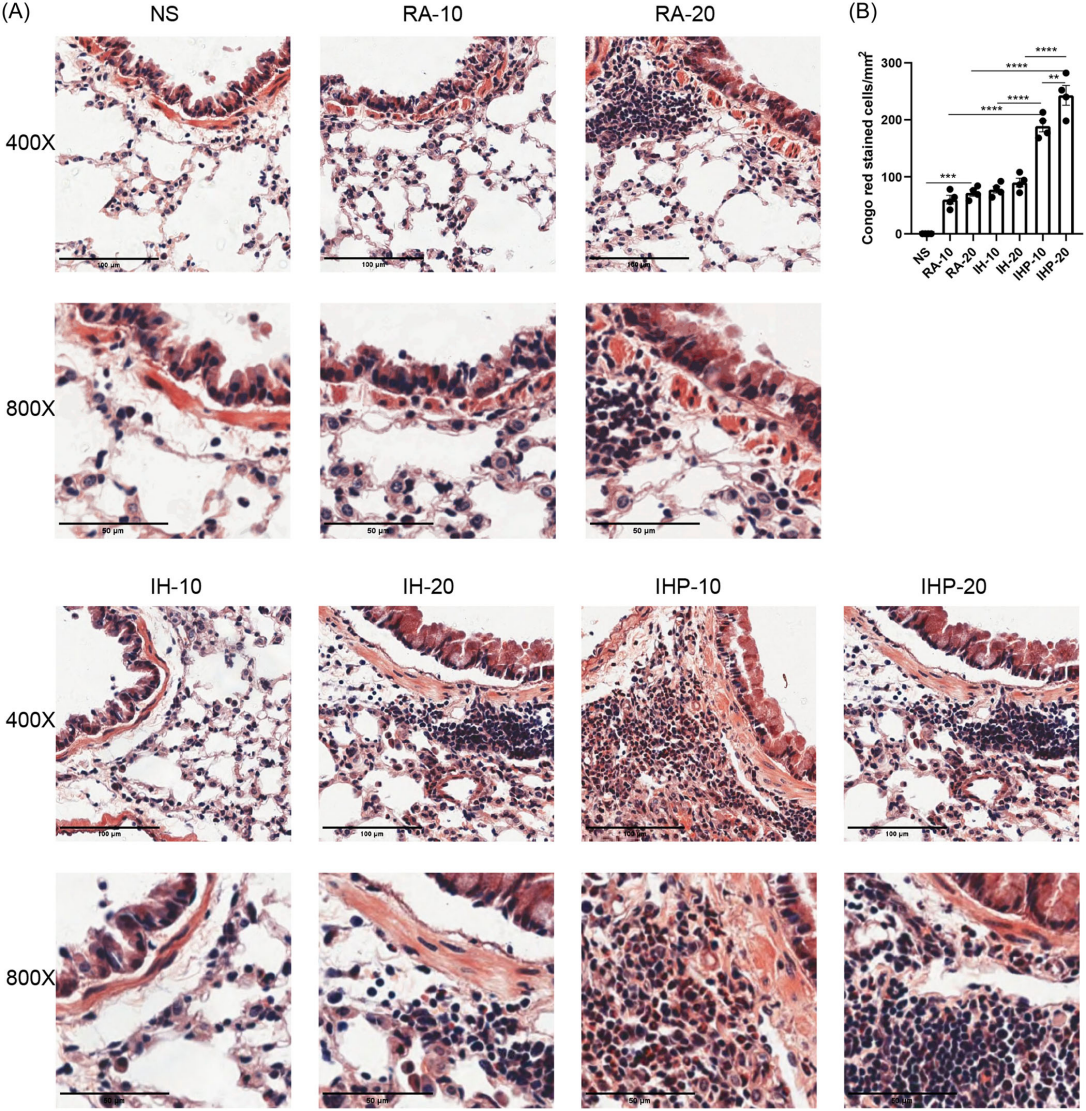
Assessment of eosinophil infiltration. (A) Numbers of Congo red+ cells per unit area (mm^2^). (B) Representative photomicrographs of Congo red stained sections of lung tissues (original magnifications ×400 and ×800). (*n* = 4 in each group) *p < .05,**p < .01, ***p < .001, ****p < .0001.

To evaluate the eosinophilic cell infiltration in mouse lung tissue, Congo red staining was performed. Results showed that compared to the control group, mice sensitized to HDM in RA exhibited an increase in eosinophilic cell aggregation around bronchioles and blood vessels (RA‐20 vs. NS, *p* < .001), while no significant difference was observed in lung tissue between the intermittent hypoxia group and the RA group (Figure 3A,B). Furthermore, following intermittent hypoxia preconditioning, the eosinophilic cell aggregation was more pronounced than in both the RA group and the intermittent hypoxia group (IHP‐20 vs. RA‐20, *p* < .0001; IHP‐20 vs. IH‐20, *p* < .0001; IHP‐10 vs. RA‐10, *p* < .0001; IHP‐10 vs. IH‐10, *p* < .0001) (Figure 3A,B).

### 3.3 The exacerbation of inflammation caused by hypoxia preconditioning is associated with NF‐κb expression, and not with mucus secretion

**Figure 4:**
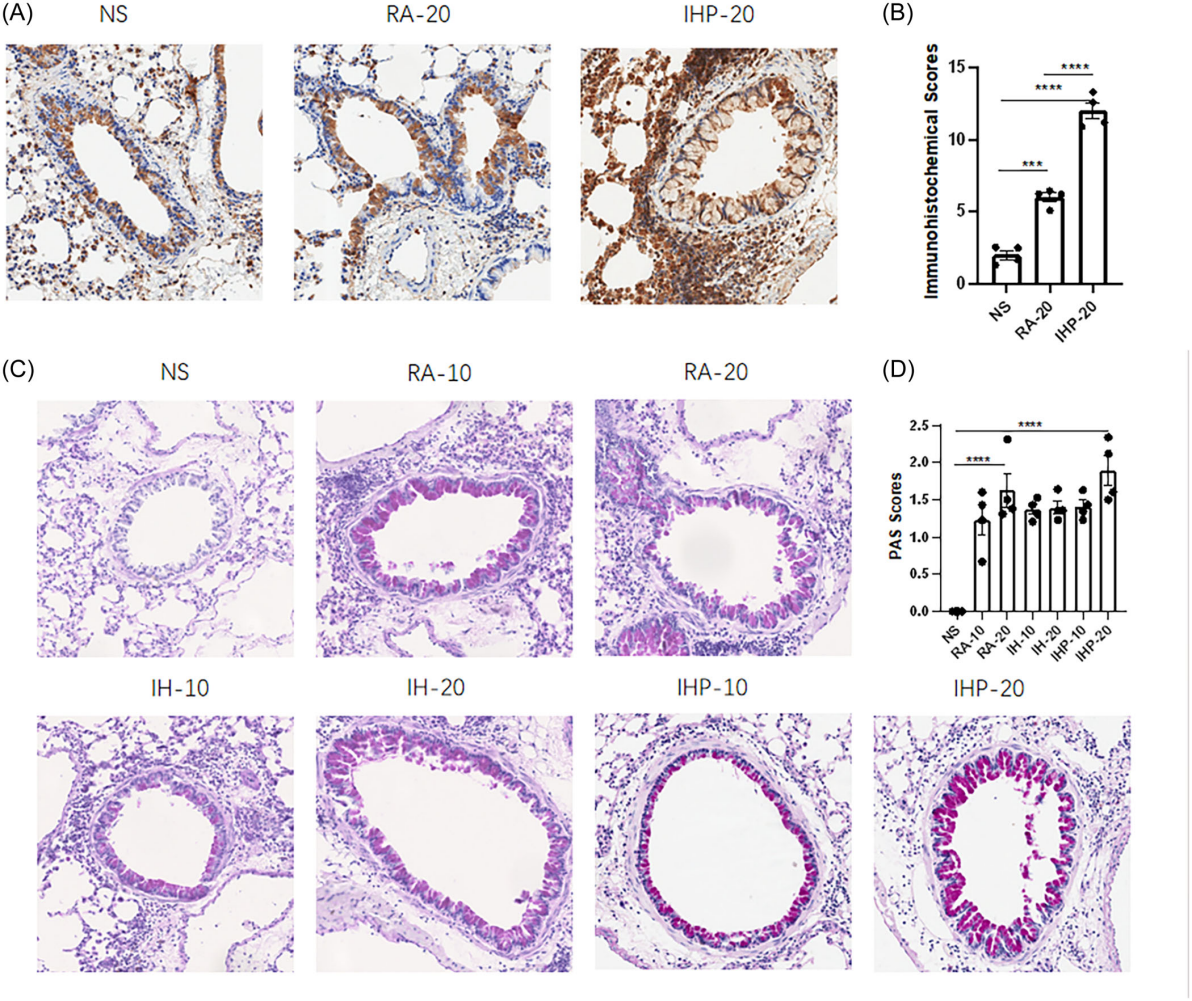
Pathological staining of lung tissue. (A) Representative photomicrographs of Lung immunohistochemistry of NF‐κB. (B) Quantitative analysis of Lung immunohistochemistry. (original magnification ×200) (C) Representative photomicrographs of PAS‐stained sections of lung tissues (original magnification ×200). (D) Mucus scores based on PAS staining. (*n* = 4 in each group) *p < .05, **p < .01, ***p < .001, ****p < .0001.

Immunohistochemical analysis and staining scores of NF‐κb showed that, compared to the control group, mice sensitized to HDM in RA exhibited higher levels of NF‐κb expression and staining scores (IH‐20 vs. NS, *p* < .05), while the intermittent hypoxia preconditioning group exhibited even more significant NF‐κb expression (IH‐20 vs. NS, *p* < .001) (Figure 4A,B). There was also a significant difference in NF‐κb expression between the intermittent hypoxia preconditioning group and the intermittent hypoxia group (IHP‐20 vs. IH‐20, *p* < .01) (Figure 4A,B). PAS staining of lung tissue showed that all HDM‐exposed mice exhibited significant mucus secretion, but the scoring results indicated no significant difference between the model groups (Figure 4C,D).

### 3.4 Intermittent hypoxia preconditioning leads to an increase in the release of pulmonary inflammatory cytokines in asthma

**Figure 5:**
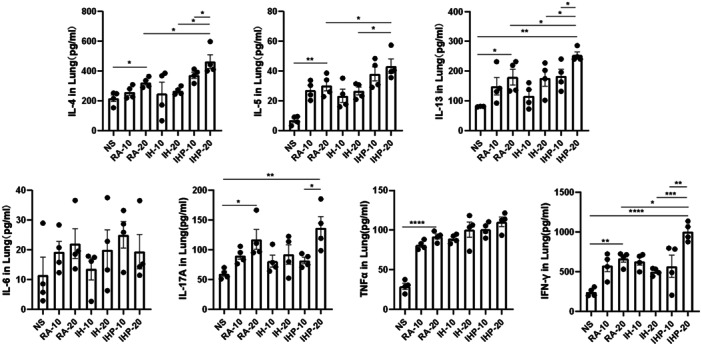
Concentrations of cytokines in lung homogenates. (*n* = 4 in each group) *p < .05, **p < .01, ***p < .001, ****p < .0001.

The findings for IL‐4, IL‐5, and IL‐13 demonstrated that mice exposed to HDM in RA had elevated levels of IL‐4 compared to the control group (RA‐20 vs. NS, *p* < .05, *p* < .01, *p* < .05, respectively) (Figure 5). There was no significant difference between the intermittent hypoxia (IH) group and the RA group for above cytokines (Figure 5). However, the intermittent hypoxia preconditioning (IHP) group exhibited a further significant increase in IL‐4, IL‐5, and IL‐13 levels compared to the RA group (IHP‐20 vs. RA‐20, *p* < .05, *p* < .05, *p* < .05, respectively), and also showed a further increase compared to the IH group (IHP‐20 vs. IH‐20, *p* < .05, *p* < .05, *p* < .05, respectively) (Figure 5).

There was no significant difference in IL‐6 levels among the groups. For IL‐17A, exposure to HDM in RA led to an increase in IL‐17A levels compared to the control group (RA‐20 vs. NS, *p* < .05), with the IHP group exhibiting a further significant increase compared to the RA group (IHP‐20 vs. NS, *p* < .01). However, no significant difference was observed between the IHP group and the IH or RA groups (Figure 5).

All groups of mice exposed to HDM showed a significant increase in TNF‐α levels compared to the control group, but there was no significant difference among the asthmatic groups. For IFN‐γ, exposure to HDM in RA led to an increase in IFN‐γ levels compared to the control group (RA‐20 vs. NS, *p* < .01) (Figure 5). There was no significant difference between the IH group and the RA group, but the IHP group exhibited a further significant increase in IFN‐γ levels compared to the RA group (IHP‐20 v.s RA‐20, *p* < .05), and also showed a further increase compared to the IH group (IHP‐20 vs. IH‐20, *p* < .001) (Figure 5).

### 3.5 IHP significantly increased asthma inflammation under high‐concentration allergen stimulation

Measurement of airway hyperresponsiveness revealed that the IHP group exhibited significantly higher Rrs when exposed to high concentration allergen stimulation after 48 mg/mL methacholine, compared to low concentration allergen stimulation (IHP‐20 vs. IHP‐10, *p* < .05). In contrast, the RA and IH groups showed only a slight increase in airway resistance under high concentration allergen stimulation compared to low concentration allergen, without any statistical difference (Figure 1A). Notably, in the IHP group, high concentration allergen stimulation resulted in increased total cells in BALF, higher HE scores, and congo scores (Figure 2B−D). These observations were not observed in the RA and IH groups. Furthermore, cytokine analysis of lung tissue samples showed a characteristic increase in IL‐4, IL‐5, IL‐13, IL‐17A, and IFN‐γ under high concentration allergen stimulation in the IHP group, while no significant changes were observed in IL‐6 and TNF‐α (Figure 4A).

## 4 DISCUSSION

Previous studies have shown that the microenvironment surrounding the site of inflammation often exhibits severe hypoxia, which can lead to pathological changes in the body.^36^ As a fundamental inflammatory airway disease, asthma is closely associated with oxygen levels. However, the exact relationship between oxygen levels and airway inflammation in asthma remains unclear. Therefore, we designed an animal model of asthma to compare the inflammatory status under different oxygen conditions, including normal oxygen, hypoxia, and pre‐hypoxia.

The data from this study showed significant difference in airway resistance (Rrs), inflammatory cell infiltration, and release of inflammatory cytokines in HDM‐induced asthma compared to NS group, however, minimal difference was observed between the asthmatic animals under hypoxia and those under normal oxygen conditions. Subsequently, we modified the oxygen delivery regimen and introduced the IHP group. Thereafter, the asthmatic animals in the pre‐hypoxia model exhibited significantly exacerbated asthma symptoms and inflammatory conditions compared to the normal oxygen and hypoxia groups, suggesting that pre‐hypoxia may worsen asthma inflammation in mice.

We then conducted a comprehensive evaluation of the inflammatory conditions in each mouse group, and the results showed that the RA and IH groups exhibited greater infiltration of eosinophils and other inflammatory cells in the airways compared to the NS group. Furthermore, the expression of various cytokines in lung tissues was increased, including Th2 cytokines (IL‐4, IL‐5, and IL‐13), Th1 cytokines (IFN‐γ and TNF‐α), and Th17 cytokines (IL‐17A).^37^ The IHP group exhibited more severe eosinophilic inflammation compared to the RA and IH groups, with a more pronounced elevation of inflammatory cytokines such as IL‐4, IL‐5, IL‐13, and IFN‐γ. IL‐5 plays a crucial role in the differentiation, recruitment, survival, and degranulation of eosinophils.^38^ The abundant production of IL‐4, IL‐5, and IL‐13 is a typical feature of type 2 asthma, and they play important roles in the generation and accumulation of eosinophils,^39^ which is consistent with the observed eosinophilic infiltration in our histopathological slices. IFN‐γ has been shown to activate eosinophils through the induction of CD69 expression.^40^ As a type 1 cytokine, IFN‐γ exerts an indirect inhibitory effect on Th2 cell differentiation, thereby suppressing IgE synthesis.^41^ This could potentially explain the less pronounced increase in IgE levels observed in the IHP group. Th17 cells are known to secrete a variety of cytokines, including IL‐17A and IL‐6, which play crucial roles in neutrophil recruitment, activation, and migration.^42^ These cytokines are considered important mediators of neutrophilic asthma.^43^ Interestingly, our study did not show a significant elevation of IL‐6 levels across the different groups. TNF‐α, which is known to induce airway inflammation and excessive mucus secretion by activating various inflammatory cells and releasing proinflammatory cytokines including IL‐6 and IL‐8, was also not significantly different in the IHP group, which may explain the lack of a significant increase in airway mucus secretion observed in the IHP group compared to the RA and IH groups. Besides, although there was a slight increase in IL‐17A levels in the IHP group compared to the RA and IH groups, the difference was not as prominent. These findings suggested that the exacerbation of inflammation induced by prehypoxia may not be strongly associated with neutrophilic infiltration, a conclusion that was supported by our histopathological examination. These results together indicated the presence of mixed immune‐mediated inflammation in the HDM‐induced mouse model of asthma, and pre‐hypoxia primarily exacerbates Th2 inflammation.

In this research, we also found that, in the IHP group, high‐concentration allergen exposure led to a more pronounced increase in inflammatory markers (such as HE scores, Congo scores, IL‐4, IL‐13, Rrs, and so on) compared to low‐concentration exposure, with statistically significant differences. However, in the RA and IH groups, high‐concentration allergen exposure only resulted in elevated levels of various inflammatory markers compared to low‐concentration exposure, with no statistical significance. These findings indicate that pre‐hypoxia treatment can amplify the asthmatic inflammatory response to high‐concentration allergen stimulation, while the correlation between inflammatory response and pre‐hypoxia treatment is weaker in the low‐concentration allergen group. Given the concentration of allergens tends to decrease as the altitude increases,^44^ studies have shown that at high altitudes, there is minimal loss of forced expiratory volume in 1 s due to physical activity and hypoxia.^45^ This phenomenon can be explained by the less impact of low oxygen on low concentration allergens, which suggests that in a hypoxic environment, the immune system becomes more sensitive to allergens, leading to a more intense inflammatory response to high‐concentration allergen exposure.

The NF‐κB signaling pathway consists of different canonical and noncanonical branches, which play complex and pleiotropic roles in inflammation control and can modulate both proinflammatory and anti‐inflammatory processes. The canonical pathway of NF‐κB signaling has been shown to respond to hypoxia.^46^ Studies have indicated a link between NF‐κB activation and asthma,^47^ and the NF‐κB pathway can regulate the inflammatory immune response in asthma through Th2 reactions.^48^ The NF‐κB pathway is considered a key regulatory pathway for modulating Th2 inflammation in allergic diseases such as asthma, involved in the activation of various inflammatory cells and the production of inflammatory mediators, such as IL‐4, IL‐5, and IL‐13.^49^ The immunohistochemical results of NF‐κB in this study suggest that preconditioning with low oxygen levels induces more severe Th2‐related inflammatory responses in asthma, and NF‐κB maybe involved in this process.

However, it is important to note that our study only examined one type of IH and IHP treatment, and we did not conduct in vitro cellular experiments. Therefore, the findings from this model are not comprehensive, and further investigation is needed to elucidate the underlying mechanisms involved.

## 5 CONCLUSION

In conclusion, our findings suggest that IHP may enhance TH2 inflammatory responses through the NF‐κB pathway, leading to exacerbated airway inflammation, increased airway hyperresponsiveness, and ultimately worsening of asthma symptoms. These results provide novel insights into the impact of a hypoxic environment on asthma development and offer a basis for developing intervention strategies targeting asthma under low oxygen conditions.

## AUTHOR CONTRIBUTIONS

**Hao Meng**: Writing—review and editing, formal analysis; investigation; project administration. **Dongxue Zhang**: Writing—review and editing; resources; methodology. **Yifan Que**: Writing—review and editing; formal analysis; methodology; data curation; **Peng Hu**: Formal analysis; methodology; software. **Runsheng Wang**: Data curation; formal analysis; methodology. **Yunfei Liao**: Con‐ceptualization; supervision; validation. **Guogang Xu**: Con‐ceptualization; writing—review and editing, supervision; validation.

## CONFLICT OF INTEREST STATEMENT

The authors declare no conflict of interest.

## ETHICS STATEMENT

All experimental procedures were approved by the Ethics Committee of Chinese PLA General Hospital (SRSW‐DWLL‐2022‐06) and in accordance with institutional guidelines for laboratory animals.

## Data Availability

The data sets used and/or analyzed during the current study are available from the corresponding author on reasonable request.
